# (*E*)-1-[(1,3-Dioxan-4-yl)meth­yl]-2-(nitro­methyl­idene)imidazolidine

**DOI:** 10.1107/S1600536810032691

**Published:** 2010-08-21

**Authors:** Zhongzhen Tian, Haijun Dong, Dongmei Li, Gaolei Wang

**Affiliations:** a Shandong Provincial Key Laboratory of Fluorine Chemistry and Chemical Materials, School of Chemistry and Chemical Engineering, University of Jinan, People’s Republic of China; b School of Sciences, University of Jinan, People’s Republic of China

## Abstract

In the title compound, C_9_H_15_N_3_O_4_, the 1,3-dioxane ring displays a chair conformation and the five-membered ring is close to planar (r.m.s. deviation = 0.054 Å). An intra­molecular N—H⋯O hydrogen bond to one of the nitro-group O atoms generates an *S*(6) ring. In the crystal, inter­molecular N—H⋯O hydrogen bonds link the mol­ecules into *C*(6) chains propagating in [010] and a C—H⋯O link also occurs.

## Related literature

For a related structure, see Tian *et al.* (2009 ▶). For background to neonicotinoid insecticides, see Mori *et al.* (2001 ▶); Ohno *et al.* (2009 ▶); Jeschke & Nauen (2008 ▶); Kagabu (1997 ▶); Tian *et al.* (2007 ▶).
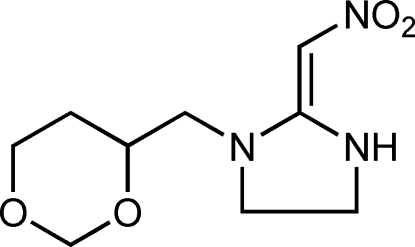


## Experimental

### 

#### Crystal data

C_9_H_15_N_3_O_4_
*M* *_r_* = 229.24
Monoclinic, 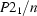

*a* = 5.0138 (4) Å
*b* = 9.8092 (9) Å
*c* = 21.7162 (18) Å
*V* = 1068.03 (16) Å^3^
*Z* = 4
Mo *K*α radiation
μ = 0.11 mm^−1^
*T* = 296 K
0.42 × 0.26 × 0.16 mm

#### Data collection

Bruker APEXII CCD diffractometer
Absorption correction: multi-scan (*SADABS*; Bruker, 2005 ▶) *T*_min_ = 0.954, *T*_max_ = 0.982
5866 measured reflections
1933 independent reflections
1395 reflections with *I* > 2σ(*I*)
*R*_int_ = 0.022

#### Refinement

*R*[*F* ^2^ > 2σ(*F* ^2^)] = 0.054
*wR*(*F* ^2^) = 0.174
*S* = 1.10
1933 reflections
145 parameters
H-atom parameters constrained
Δρ_max_ = 0.35 e Å^−3^
Δρ_min_ = −0.28 e Å^−3^

### 

Data collection: *APEX2* (Bruker, 2005 ▶); cell refinement: *SAINT* (Bruker, 2005 ▶); data reduction: *SAINT*; program(s) used to solve structure: *SIR97* (Altomare *et al.*, 1999 ▶); program(s) used to refine structure: *SHELXL97* (Sheldrick, 2008 ▶); molecular graphics: *SHELXTL* (Sheldrick, 2008 ▶); software used to prepare material for publication: *WinGX* (Farrugia, 1999 ▶).

## Supplementary Material

Crystal structure: contains datablocks I, global. DOI: 10.1107/S1600536810032691/hb5607sup1.cif

Structure factors: contains datablocks I. DOI: 10.1107/S1600536810032691/hb5607Isup2.hkl

Additional supplementary materials:  crystallographic information; 3D view; checkCIF report

## Figures and Tables

**Table 1:** Hydrogen-bond geometry (Å, °)

*D*—H⋯*A*	*D*—H	H⋯*A*	*D*⋯*A*	*D*—H⋯*A*
N2—H2⋯O2	0.86	2.17	2.694 (3)	119
N2—H2⋯O1^i^	0.86	2.17	2.824 (3)	133
C1—H1⋯O2^ii^	0.93	2.42	3.249 (3)	148

Symmetry codes: (i) 
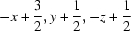
; (ii) 
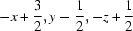
.

**Fig. 1.**
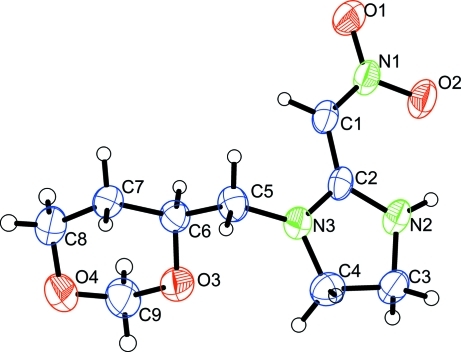
The molecular structure of (I) with displacement ellipsoids drawn at the 40% probability level. The H atoms are shown as spheres of arbitrary size.

## supplementary crystallographic information

### Comment

By virtue of novel modes of action (targeting insect nicotinic acetylcholine receptors (nAChRs) (Ohno *et al.*, 2009), low mammalian toxicity, broad insecticidal spectra, and good systemic properties (Jeschke *et al.*, 2008), neonicotinoids has accounted for 18% of world insecticide sales in the past decades. Our interest was introducing oxygen atoms into the lead struture and synthesizing a series of new compounds, in which the title compound exhibited moderate insecticidal activities against pea aphids.

The structure of the title compound is shown in Fig. 1 with the atom-numbering scheme. The 1,3-dioxane ring displays an chair conformation with bond angles lying between 110.0 (2)° and 111.7 (2)°. The nitro moiety is in *trans* configuration relative to the 1,3-dioxane ring and coplanar with the olefin-amine plane [N3—C2—C1—N1 = -177.49 (18)°]. Around N2 and N3 atoms the sums of the angles are 360° and 359.72°, respectively, indicating that they are typical *sp*^2^ hybridized and leading to an essentially planar imidazole ring.

### Experimental

A solution of *N*-((1,3-dioxan-4-yl)methyl)ethane-1,2-diamine (2 mmol), and 1,1-bis(thiomethyl)-2-nitroethylene (2 mmol) in 30 ml of ethanol was refluxed for 8 h and then cooled to room temperature. Evaporation under reduced pressure gave the title product after purifiction by flash chromatography.  Colourless prisms of (I) were obtained by slow evaporation of a solution of the title compound in dichloromethane and ethyl acetate.

### Refinement

All H atoms were placed in their calculated positions and then refined using riding model with C—H = 0.93–0.99 Å, *U*_iso_(H) = 1.2 (1.5 for methyl groups) times *U*_eq_(C).

### Crystal data

**Table:**

C_9_H_15_N_3_O_4_	*F*(000) = 488
*M**_r_* = 229.24	*D*_x_ = 1.426 Mg m^−^^3^
Monoclinic, *P*2_1_/*n*	Mo *K*α radiation, λ = 0.71073 Å
Hall symbol: -P 2yn	Cell parameters from 2831 reflections
*a* = 5.0138 (4) Å	θ = 3.5–28.7°
*b* = 9.8092 (9) Å	µ = 0.11 mm^−^^1^
*c* = 21.7162 (18) Å	*T* = 296 K
β = 90°	Prism, colourless
*V* = 1068.03 (16) Å^3^	0.42 × 0.26 × 0.16 mm
*Z* = 4	

### Data collection

**Table:**

Bruker APEXII CCD diffractometer	1933 independent reflections
Radiation source: fine-focus sealed tube	1395 reflections with *I* > 2σ(*I*)
graphite	*R*_int_ = 0.022
φ and ω scans	θ_max_ = 25.4°, θ_min_ = 3.5°
Absorption correction: multi-scan (*SADABS*; Bruker, 2005)	*h* = −6→6
*T*_min_ = 0.954, *T*_max_ = 0.982	*k* = −11→11
5866 measured reflections	*l* = −26→26

### Refinement

**Table:**

Refinement on *F*^2^	Primary atom site location: structure-invariant direct methods
Least-squares matrix: full	Secondary atom site location: difference Fourier map
*R*[*F*^2^ > 2σ(*F*^2^)] = 0.054	Hydrogen site location: inferred from neighbouring sites
*wR*(*F*^2^) = 0.174	H-atom parameters constrained
*S* = 1.10	*w* = 1/[σ^2^(*F*_o_^2^) + (0.1126*P*)^2^] where *P* = (*F*_o_^2^ + 2*F*_c_^2^)/3
1933 reflections	(Δ/σ)_max_ < 0.001
145 parameters	Δρ_max_ = 0.35 e Å^−^^3^
0 restraints	Δρ_min_ = −0.28 e Å^−^^3^

### Special details

**Table:**

Geometry. All e.s.d.'s (except the e.s.d. in the dihedral angle between two l.s. planes) are estimated using the full covariance matrix. The cell e.s.d.'s are taken into account individually in the estimation of e.s.d.'s in distances, angles and torsion angles; correlations between e.s.d.'s in cell parameters are only used when they are defined by crystal symmetry. An approximate (isotropic) treatment of cell e.s.d.'s is used for estimating e.s.d.'s involving l.s. planes.
Refinement. Refinement of *F*^2^ against ALL reflections. The weighted *R*-factor *wR* and goodness of fit *S* are based on *F*^2^, conventional *R*-factors *R* are based on *F*, with *F* set to zero for negative *F*^2^. The threshold expression of *F*^2^ > σ(*F*^2^) is used only for calculating *R*-factors(gt) *etc*. and is not relevant to the choice of reflections for refinement. *R*-factors based on *F*^2^ are statistically about twice as large as those based on *F*, and *R*- factors based on ALL data will be even larger.

### Fractional atomic coordinates and isotropic or equivalent isotropic displacement parameters (Å^2^)

**Table:**

	*x*	*y*	*z*	*U*_iso_*/*U*_eq_	
O2	0.7955 (3)	0.71286 (16)	0.23056 (8)	0.0532 (5)	
N1	0.7422 (4)	0.58813 (19)	0.22961 (9)	0.0420 (5)	
C2	0.3945 (4)	0.6124 (2)	0.30617 (10)	0.0343 (5)	
O3	0.2691 (4)	0.39753 (16)	0.44986 (7)	0.0536 (5)	
N2	0.4042 (4)	0.74655 (19)	0.31417 (9)	0.0435 (5)	
H2	0.5038	0.8003	0.2930	0.052*	
C1	0.5501 (4)	0.5344 (2)	0.26581 (10)	0.0390 (6)	
H1	0.5196	0.4409	0.2638	0.047*	
N3	0.2104 (4)	0.55718 (19)	0.34370 (8)	0.0421 (5)	
O1	0.8720 (4)	0.51016 (18)	0.19406 (9)	0.0640 (6)	
C5	0.1287 (5)	0.4153 (2)	0.34624 (11)	0.0432 (6)	
H5A	0.1514	0.3753	0.3057	0.052*	
H5B	−0.0597	0.4115	0.3563	0.052*	
C6	0.2786 (5)	0.3314 (2)	0.39185 (10)	0.0406 (6)	
H6	0.4653	0.3268	0.3787	0.049*	
O4	0.2921 (5)	0.1954 (2)	0.50513 (9)	0.0741 (7)	
C7	0.1743 (5)	0.1882 (2)	0.39737 (11)	0.0453 (6)	
H7A	0.2109	0.1387	0.3596	0.054*	
H7B	−0.0175	0.1904	0.4032	0.054*	
C4	0.0734 (5)	0.6627 (3)	0.37948 (11)	0.0474 (6)	
H4B	0.0835	0.6438	0.4233	0.057*	
H4A	−0.1125	0.6699	0.3675	0.057*	
C3	0.2257 (5)	0.7920 (2)	0.36326 (11)	0.0507 (7)	
H3A	0.1061	0.8629	0.3488	0.061*	
H3B	0.3255	0.8258	0.3984	0.061*	
C9	0.4109 (7)	0.3220 (3)	0.49462 (13)	0.0653 (8)	
H9A	0.5930	0.3086	0.4808	0.078*	
H9B	0.4163	0.3731	0.5328	0.078*	
C8	0.3037 (7)	0.1152 (3)	0.45115 (13)	0.0651 (8)	
H8B	0.2132	0.0293	0.4583	0.078*	
H8A	0.4885	0.0956	0.4413	0.078*	

### Atomic displacement parameters (Å^2^)

**Table:**

	*U*^11^	*U*^22^	*U*^33^	*U*^12^	*U*^13^	*U*^23^
O2	0.0554 (11)	0.0286 (10)	0.0756 (13)	−0.0030 (7)	0.0114 (9)	0.0037 (8)
N1	0.0450 (11)	0.0278 (11)	0.0533 (12)	0.0051 (8)	0.0010 (9)	0.0052 (9)
C2	0.0377 (11)	0.0255 (12)	0.0397 (12)	0.0020 (9)	−0.0111 (9)	0.0038 (9)
O3	0.0719 (11)	0.0445 (11)	0.0443 (10)	−0.0009 (9)	−0.0041 (8)	−0.0026 (8)
N2	0.0474 (11)	0.0262 (10)	0.0569 (12)	0.0006 (8)	0.0004 (9)	0.0001 (9)
C1	0.0458 (13)	0.0261 (12)	0.0451 (13)	−0.0020 (9)	−0.0025 (10)	0.0019 (10)
N3	0.0524 (12)	0.0317 (11)	0.0423 (11)	−0.0025 (8)	0.0010 (9)	0.0015 (8)
O1	0.0772 (13)	0.0398 (11)	0.0750 (13)	0.0116 (9)	0.0302 (10)	0.0017 (10)
C5	0.0450 (13)	0.0410 (14)	0.0437 (13)	−0.0091 (11)	−0.0063 (10)	0.0035 (10)
C6	0.0441 (12)	0.0376 (13)	0.0399 (12)	−0.0056 (10)	−0.0015 (9)	0.0002 (10)
O4	0.1233 (19)	0.0549 (12)	0.0441 (10)	−0.0022 (12)	0.0030 (11)	0.0089 (9)
C7	0.0536 (14)	0.0350 (13)	0.0471 (14)	−0.0038 (10)	0.0041 (11)	−0.0019 (10)
C4	0.0462 (13)	0.0482 (15)	0.0477 (13)	0.0052 (11)	−0.0027 (10)	−0.0031 (12)
C3	0.0677 (17)	0.0349 (14)	0.0494 (14)	0.0088 (12)	−0.0027 (12)	−0.0024 (11)
C9	0.095 (2)	0.0565 (18)	0.0446 (15)	0.0001 (15)	−0.0174 (14)	0.0038 (13)
C8	0.096 (2)	0.0411 (16)	0.0584 (17)	0.0051 (15)	0.0003 (15)	0.0049 (13)

### Geometric parameters (Å, °)

**Table:**

O2—N1	1.253 (2)	C6—H6	0.9800
N1—C1	1.350 (3)	C6—C7	1.504 (3)
N1—O1	1.267 (2)	O4—C9	1.396 (4)
C2—N2	1.329 (3)	O4—C8	1.413 (3)
C2—C1	1.401 (3)	C7—H7A	0.9700
C2—N3	1.345 (3)	C7—H7B	0.9700
O3—C6	1.418 (3)	C7—C8	1.516 (4)
O3—C9	1.414 (3)	C4—H4B	0.9700
N2—H2	0.8600	C4—H4A	0.9700
N2—C3	1.462 (3)	C4—C3	1.522 (4)
C1—H1	0.9300	C3—H3A	0.9700
N3—C5	1.452 (3)	C3—H3B	0.9700
N3—C4	1.465 (3)	C9—H9A	0.9700
C5—H5A	0.9700	C9—H9B	0.9700
C5—H5B	0.9700	C8—H8B	0.9700
C5—C6	1.491 (3)	C8—H8A	0.9700
			
O2—N1—C1	121.61 (19)	H5A—C5—H5B	107.6
O2—N1—O1	119.35 (19)	C6—C5—H5A	108.7
N1—C1—C2	123.2 (2)	C6—C5—H5B	108.7
N1—C1—H1	118.4	C6—C7—H7A	109.5
C2—N2—H2	124.0	C6—C7—H7B	109.5
C2—N2—C3	112.0 (2)	C6—C7—C8	110.7 (2)
C2—C1—H1	118.4	O4—C9—O3	111.3 (2)
C2—N3—C5	127.03 (19)	O4—C9—H9A	109.4
C2—N3—C4	111.03 (19)	O4—C9—H9B	109.4
O3—C6—C5	108.72 (19)	O4—C8—C7	111.0 (2)
O3—C6—H6	108.2	O4—C8—H8B	109.4
O3—C6—C7	110.17 (18)	O4—C8—H8A	109.4
O3—C9—H9A	109.4	C7—C6—H6	108.2
O3—C9—H9B	109.4	C7—C8—H8B	109.4
N2—C2—C1	127.1 (2)	C7—C8—H8A	109.4
N2—C2—N3	110.1 (2)	H7A—C7—H7B	108.1
N2—C3—C4	102.82 (18)	C4—C3—H3A	111.2
N2—C3—H3A	111.2	C4—C3—H3B	111.2
N2—C3—H3B	111.2	H4B—C4—H4A	109.1
N3—C2—C1	122.80 (19)	C3—N2—H2	124.0
N3—C5—H5A	108.7	C3—C4—H4B	111.1
N3—C5—H5B	108.7	C3—C4—H4A	111.1
N3—C5—C6	114.35 (18)	H3A—C3—H3B	109.1
N3—C4—H4B	111.1	C9—O3—C6	110.8 (2)
N3—C4—H4A	111.1	C9—O4—C8	110.0 (2)
N3—C4—C3	103.34 (19)	H9A—C9—H9B	108.0
O1—N1—C1	119.04 (19)	C8—C7—H7A	109.5
C5—N3—C4	121.66 (19)	C8—C7—H7B	109.5
C5—C6—H6	108.2	H8B—C8—H8A	108.0
C5—C6—C7	113.16 (18)		
			
O2—N1—C1—C2	0.9 (3)	N3—C5—C6—O3	52.3 (3)
C2—N2—C3—C4	−7.0 (2)	N3—C5—C6—C7	175.06 (19)
C2—N3—C5—C6	92.4 (3)	N3—C4—C3—N2	7.6 (2)
C2—N3—C4—C3	−6.3 (2)	O1—N1—C1—C2	−179.4 (2)
O3—C6—C7—C8	−49.0 (3)	C5—N3—C4—C3	179.39 (19)
N2—C2—C1—N1	1.8 (3)	C5—C6—C7—C8	−170.9 (2)
N2—C2—N3—C5	176.06 (19)	C6—O3—C9—O4	−64.4 (3)
N2—C2—N3—C4	2.1 (2)	C6—C7—C8—O4	49.6 (3)
C1—C2—N2—C3	−176.0 (2)	C4—N3—C5—C6	−94.3 (3)
C1—C2—N3—C5	−4.5 (3)	C9—O3—C6—C5	−179.5 (2)
C1—C2—N3—C4	−178.49 (19)	C9—O3—C6—C7	56.0 (3)
N3—C2—N2—C3	3.4 (2)	C9—O4—C8—C7	−56.4 (3)
N3—C2—C1—N1	−177.49 (18)	C8—O4—C9—O3	64.1 (3)

### Hydrogen-bond geometry (Å, °)

**Table:**

*D*—H···*A*	*D*—H	H···*A*	*D*···*A*	*D*—H···*A*
N2—H2···O2	0.86	2.17	2.694 (3)	119
N2—H2···O1^i^	0.86	2.17	2.824 (3)	133
C1—H1···O2^ii^	0.93	2.42	3.249 (3)	148

Symmetry codes: (i) −*x*+3/2, *y*+1/2, −*z*+1/2; (ii) −*x*+3/2, *y*−1/2, −*z*+1/2.
